# The Association of Aquaporin-1 Gene with Marathon Running Performance Level: a Confirmatory Study Conducted in Male Hispanic Marathon Runners

**DOI:** 10.1186/s40798-020-00243-0

**Published:** 2020-03-20

**Authors:** Miguel A. Rivera, Thomas D. Fahey, Juan R. López-Taylor, Juan L. Martínez

**Affiliations:** 1 Department of Physical Medicine, Rehabilitation & Sports Medicine, School of Medicine, University of Puerto Rico, Main Building Office A204, San Juan, PR 00936 USA; 2 Department of Kinesiology, California State University, Chico, CA USA; 3 Physical Activity and Applied Sport Sciences Institute, Universidad de Guadalajara, Guadalajara, Jalisco México; 4 Hospital Civil de Culiacán, Culiacán, Sinaloa México

**Keywords:** Meta-analysis, Genetic epidemiology, Marathon, Humans, Men, Cochran’s *Q* test, Exercise

## Abstract

**Background:**

Replication studies are essential for identifying credible associations between alleles and phenotypes. Validation of genotype-phenotype associations in the sports and exercise field is rare. An initial genetic association study suggested that rs1049305 (C > G) in the 3′ untranslated region (3′UTR) of the aquaporin-1 (AQP1) gene was associated with marathon running (MR) performance level in Hispanic males. To validate this finding, we conducted a replication analysis in an independent case-control sample of Hispanic male marathon runners (*n* = 1430; cases *n* = 713 and controls *n* = 717). A meta-analysis was utilized to test the extent of the association between the initial results and the present report. It also provided to test the heterogeneity (variation) between the two studies.

**Results:**

The replication study showed a statistically significant (*p* ≤ 0.05) association between rs1049305 (C > G) of the AQP1 gene and MR performance level. Association test results using a fixed effect model for the combined, original study and the present report, yielded an odds ratio = 1.28, 95% confidence interval = 1.13–1.45, *p* = 0.0001. The extent of the measures of heterogeneity was Tau-squared = 0, *H* statistic = 1, *I*^2^ statistic = 0, and Cochran’s *Q* test (*Q* = 0.29; *p* value 0.59), indicated the variation between studies were due to chance and not to differences in heterogeneity between the two studies. Within the limitations of the present replication, contrast of two studies and its effects on meta-analysis, the findings were robust.

**Conclusion:**

This study successfully replicated the results of Martínez et al. (Med Sportiva 13:251-5, 2009). The meta-analysis provided further epidemiological credibility for the hypothesis of association between the DNA rs1049305 (C > G) variation in the 3′UTR of the AQP1 gene and MR running performance level in Hispanics male marathon runners. It is not precluded that a linked DNA structure in the surrounding molecular neighborhood could be of influence by been part of the overly complex phenotype of MR performance level.

## Key Points

- Replication studies of genotype-phenotype associations in the sports and exercise field literature are rare.
- Replication of findings from genetic association studies is a fundamental assessment process in research endeavors leading to new knowledge.
- *AQP1* gene coexistent molecular mechanisms and their effects on marathon running performance phenotypes are major areas of inquiry in the science and medicine of sport and exercise.

## Introduction

Aquaporins (AQPs) are integral membrane pore proteins, also known as water channels. The aquaporin-1 (AQP1) channel is the best known and most studied of the AQP family [1]. The AQP1 channel is encoded by the aquaporin-1 (AQP1) gene, on chromosome 7, region p14 [2]. The expression of AQP1 occurs in various tissues, including red blood cells, endothelial cells, and smooth, skeletal, and cardiac muscle [1, 3]. The major AQP of the cardiovascular system is AQP1, which regulates water permeability of the heart’s capillary networks by mediating the flow of water through the endothelial layer into the blood.

Evidence from a recent systematic review [4] on the association between AQP1 and endurance performance (EP) indicated that the first effort to evaluate the hypothesis of association between the AQP1 gene DNA sequence variant rs1049305 (C > G) in the 3′ untranslated region (3′UTR) and marathon running (MR) performance level was conducted by Martinez et al. [5]. Genotypic and allelic frequency distribution revealed significant differences (*X*^2^, *P* = 0.005) between cases (fast marathon runners–the top three % of finishers) and controls (slow marathon runners–the low three % of finishers). The calculated odds ratio (OR) = 1.35. Its 95% confidence interval boundary was 1.08 to 1.67. The C-allele was more (*P* = 0.005) prevalent in the cases (i.e., fast runners) than in the controls (slow runners). To date, these data have not been replicated.

The confirmation of a genetic-association finding is vital to identifying true positive genetic variants that may contribute to complex phenotypes, such as MR performance level [6] To confirm findings and make generalizations, the reproduction of results from genetic-association studies should occur using independent samples [7]. Along those lines, an independent study [8] confirmed the original findings of the association between MR performance level and the rs1049305 (C > G), variant within the 3′UTR of the AQP1 gene. This time, the association was evaluated in South African Caucasian male Ironman triathletes (*n* = 504). The findings revealed that the AQP1 rs1049305 C-variant was associated with the duration of the MR segment in three Ironman events. Triathletes who carried the C-allele completed the 42.2-km run stage faster (mean 286, *s* = 49 min) than triathletes with the GG genotype (mean 296, *s* = 47 min; *P* = 0.032).

The same association was found in another observational study using a genetic epidemiology model [9]. In that study, elapsed running time in a 10-km event was compared by AQP1 C-allele carrier status, e.g., carriers (homozygous for C-allele (CC) and heterozygous for C-allele (CG); *n* = 50) versus non-carriers (homozygous for G-allele (GG); *n* = 41). That study revealed that AQP1 C-allele carries ran faster (*P* < 0.05) than non-carriers during the 10-km race. Those findings to a certain extent validated [10] the association between endurance running performance and the rs1049305 (C > G) variant within the 3′UTR region of the AQP1 gene. The 3′-UTR is a significant participant in regulating gene expression by controlling nuclear export, subcellular targeting, and rates of translation and degradation of DNA. These functions make the 3′UTR an essential tool for regulating phenotypic diversity of in humans [11]. The genes controlled by the sequence of the 3′UTR are generally regulatory proteins, and their irregular expression may have severe effects on humans [11, 12].

To date, no exact replication [10, 13] of the first reported association [5] between the rs1049305 (C > G), in the 3′UTR of the AQP1 gene and MR performance level in Hispanic male marathon runners that has been reported. The concept of exact replication implies the examination of the same genetic variant in an independent sample drawn from the same population as the initial study, using a similar design and methods [10, 13]. The related literature rarely evidence exact replication studies examining the association of genetic markers and MR performance level using comparable subjects, similar methods, and procedures as those of the original publications. Therefore, the purposes of the present research were (1) to verify the hypothesis of association between the AQP1 rs1049305 (C > G) genetic marker and CE MR performance level in Hispanic male marathon runners [5] and (2) to exactly replicate the original study by Martinez et al. [5]. We adhered to established criteria [14, 15] and gathered a larger sample size (adequate statistical power), recruited, screened, and evaluated in an identical approach as that of the initial study [5].

## Methods

This observational study adheres to a genetic epidemiology model using a case-control design. All methods and procedures were identical to those described by Martinez et al. [5].

### Subjects

The subjects in the present study (*n* = 1430; cases *n* = 713 and controls *n* = 717) were male biologically unrelated volunteers, recruited during the activities of marathon (42 km) running events conducted between 2006 and 2012: Maratón de Culiacan, Gran Maratón Pacifico, Mazatlán, and Guadalajara International Marathon all held in México. The physical characteristics of the cases and controls are shown in Table 1.

**Table 1:** Characteristics of 1430 biologically unrelated marathon runners in the present replication study

Group	Age (years)	Height (cm)	Mass (kg)
Cases (*n* = 713)	32 ± 18	173 ± 15	62 ± 7
Controls (*n* = 717)	39 ± 19	175 ± 11	68 ± 12

Values are mean ± standard deviation

The recruitment process included an interview in determining the ethnic and geographic area of origin, place of residence, history of endurance events participation, cardiovascular disease, respiratory disease, arthritis activity, history of metabolic disease, and the use of related medications.

### Exclusion Criteria

Subjects with a history of cardiovascular, respiratory, arthritis, metabolic disease, and taking-related medications were excluded. Subjects reporting any incident that negatively affected the performance during the race (e.g., significant injury) were also excluded. Subjects that participated in the initial study [5] were also excluded from the analysis.

### Subjects’ Classification

The official results of the competitions determined the classification as cases or controls. Cases (*n* = 713) were the top 3% of finishers of their respective age group, while controls (*n* = 717) were the lowest 3% of their respective age group.

### Ethical Standards

The study was carried out following the World Medical Association Declaration of Helsinki ethical principles for research involving human subjects [16]. The protocol of the study was approved by the Committee on Human Research of the Instituto Sinaloence del Deporte in Culiacan, Mexico and the Medical Commissions from each competition. Each participant provided a voluntary written informed consent to be part of the study.

### DNA Isolation and Genotype Determination

The Promega Wizard Genomic DNA Purification Kit (Promega Corporation, Madison, WI) provided for the purification of genomic DNA from 10 mL of whole blood. The detailed procedures were those in the original study [5]. The amplification of the AQP1 polymorphism (restriction site (rs) #1049305) followed according to the previous procedure [5]. A systematic random sample of DNA aliquots followed the completion of all the genotyping analyses. The selected DNA samples were submitted to a new round of PCR and genotyping, as described in the original study [5]. We performed a new genotyping cycle analysis if a mismatch occurred between the first and second genotyping. All work (DNA extraction, storage and management, and PCR and genotyping) were performed at the facilities of the Exercise Physiology and Human Genomics Laboratory of the Department of Physical Medicine, Rehabilitation and Sports Medicine at the School of Medicine of the University of Puerto Rico, San Juan.

### Statistical Analysis

The statistical analysis of the actual replication study was identical to those in the original study [5]. The polymorphism information content (PIC) and the heterozygosity index (*H*_I_), both measures of polymorphism, followed the procedures described [17, 18]. The Pearson chi-square test was used to examine differences in genotype and allele frequencies between cases and controls and to determine whether the genotypes prevalence distribution differed from that expected under the Hardy-Weinberg equilibrium (HWE). Odds ratios with 95% confidence interval and the chi-square tests were performed using the Epi Info™ 3.5.1 software package [19], which was the software application utilized in the original study [5]. The present and original study statistical analysis were corroborated using the most recent Epi Info™ software package version 7.2.2.6 released on February 2, 2018. A meta-analysis [20] was performed to test the extent of the association between the results of Martinez et al. [5] and the present report and to test for heterogeneity between the two studies. Heterogeneity (*H*) in meta-analysis refers to the variation in study outcomes between studies. *P* values ≤ 0.05 were statistically significant.

## Results

### Subjects

The present replication study involved 1430 subjects (cases *n* = 713 and controls *n* = 717) while the initial study by Martinez et al. [5] included 784 subjects (cases *n* = 396 and controls *n* = 388). This replication study used 646 more subjects than the original study, which reduced the probability of a type II error.

### Genotype Frequencies

As shown in Table 2, the three expected AQP1 rs1049305 genotypes (CC, CG, GG) were observed in both cases and control groups. These genotype frequencies agreed with those expected under HWE (*X*^2^, *P* ≥ 0.05). chi-square test revealed a statistically significant (*p* = 0.02) difference in the genotype frequency distribution between the cases and controls. The present result replicates and validates the original study [5], see Table 3, and corroborates those of others [8, 9].

**Table 2:** Allele and genotype frequencies of the *Aquaporin-1* gene rs1049305 (C > G) variant within the 3′ untranslated region in cases (fast marathon runners) and controls (slow marathon runners) in the present replication study

	^c^Genotype frequencies			^d^Allele frequencies				
	CC (%)	CG (%)	GG (%)	C (%)	G (%)	Odds ratio	95% Confidence interval	*P value*
^a^Cases (*n* = 713)	107 (0.15)	314 (0.44)	292 (0.41)	528 (0.37)	898 (0.63)			
^b^Controls (*n* = 717)	79 (0.11)	301 (0.42)	337 (0.47)	459 (0.34)	547 (0.66)	1.43	1.21–1.68	< 0.05

^a^Hardy-Weinberg equilibrium = 2.1, *P* = 0.14

^b^Hardy-Weinberg equilibrium = 0.9, *P* = 0.34

^c^*X*^2^ = 7.7, *df* = 2, *P* = 0.02

^d^*X*^2^ = 17.7, *df* = 1, *P* < 0.001

**Table 3:** Allele and genotype frequencies of the *Aquaporin-1* gene rs1049305 (C > G) variant within the 3′ untranslated region in cases (fast marathon runners) and controls (slow marathon runners) as reported in the original study [5]

	^c^Genotype frequencies			^d^Allele frequencies				
	CC (%)	CG (%)	GG (%)	C (%)	G (%)	Odds ratio	95% Confidence interval	*P* value
^a^Cases (*n* = 396)	59 (0.15)	167 (0.42)	170 (0.43)	285 (0.36)	506 (0.64)			
^b^Controls (*n* = 388)	39 (0.10)	151 (0.39)	198 (0.51)	229 (0.30)	547 (0.70)	1.35	1.08–1.67	< 0.005

^a^Hardy-Weinberg equilibrium = 0.09

^b^Hardy-Weinberg equilibrium = 0.21

^c^*X*^2^ = 6.94, *df* = 2, *P* = 0.03

^d^*X*^2^ = 7.55 *df* = 1, *P* = 0.005

### Allelic Frequencies

Table 2 also shows the observed allelic frequency distribution in cases and controls. The allelic frequency distributions revealed statistically significant (*P* < 0.001) differences between the cases and controls. The C-allele was the less frequently observed in both cases and controls.

### Allelic Frequencies by C-allele Carrier Status

In the present study, the prevalence of C-allele carrier status was 59% (107 + 314/713) for the cases and 53% (79 + 301/717) for the controls. The calculated odds ratio (OR = 1.43) and its 95% confidence interval (1.21–1.68), suggested that the C-allele was more likely prevalent (*p* < 0.05) in the cases than in the controls. In the original study, 57% of the cases were carriers of the C-allele versus 49% for the controls. The computed OR = 1.35 and its 95% confidence interval extended from 1.08 to 1.67; *p* < 0.05. These findings also replicated and validated those of the original study [5].

### AQP1 Polymorphisms

Table 4 shows the calculated values of the *H*_I_ and PIC for the original study [5] and those of the present report were the same for both measures of polymorphism. These data replicate the findings of the original study. *H*_I_ is a (expected) probability measure of the likelihood that an individual will be heterozygous at the AQP1 rs1049305. Also, *H*_I_ is a central measure in the study of genetic variation in populations. For two allele loci, like the one represented by the AQP1 gene rs1049305 (C > G), *H*_I_ peaks at a value of 0.5.

**Table 4:** Heterozygosity (*H*_I_) and polymorphism information content (PIC) from original [5] and present exact replication studies

Group		Original study	Present study
Cases	*H*_I_	0.46	0.47
	PIC	0.35	0.36
Controls	*H*_I_	0.42	0.43
	PIC	0.33	0.34

PIC value is an indicator of the probability that a marker locus (AQP1 rs1049305) is informative. It is estimated from the frequency of heterozygotes, determined by the number of marker alleles (allele C and allele G), and their respective frequencies.

**Fig. 1:**
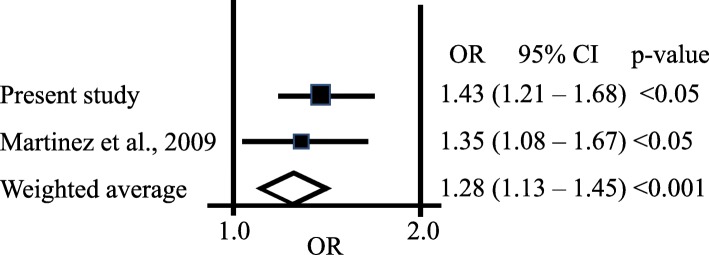
Forest plot showing the results of a meta-analysis for the association between the C > G (rs1049305) variant within the 3′ untranslated region of the *Aquaporin 1* gene in cases (fast marathon runners) and controls (slow marathon runners) as reported in the original study by Martinez et al. [5] and the present replication study. Each black square represents the odds ratios and the horizontal lines denote the 95% confidence interval for each of the identified sources. The diamond symbol shows the meta-analysis cumulative fixed effect odds ratio (OR = 1.28) for the association of the combined data of Martinez et al. [5] and the actual replication. The width of the diamond refers to the 95% confidence interval (95% CI = 1.13–1.45), *p* value < 0.001

Table 5 shows the meta-analysis association that combined data from Martinez et al. [5] and the present study. The findings in Table 5 are the outcome of a fixed effect model (FE). A principal assumption of FE meta-analysis is that the effect size is alike within the studies examined by the meta-analysis. The unadjusted and adjusted and *p* values, 0.0001 and 0.0007, for the combined FE association test were highly significant. This finding affirms the association between the AQP1 gene rs1049305 (C > G) and MR performance level. Figure 1 illustrates a forest plot showing the present findings, the findings of the original study, and the combine results of the meta-analysis for the association between the AQP1 gene rs1049305 (C > G) and MR performance.

**Table 5:** Meta-analysis association test results for the combined study of Martinez et al. [5] and the present report

Model	OR	95% CI	*p* value	adjusted *p* value
Fixed effect	1.28	1.13–1.45	0.0001	0.0007

### Meta-Analysis Test of Heterogeneity

The meta-analysis also provided four indicators of heterogeneity (variation) between the two studies under consideration. The extent of these four measures of heterogeneity is shown in Table 6.

**Table 6:** Test of heterogeneity (variation) results between the study of Martinez et al. [5] and the present report

Tau-squared (tau^2^)	*H* statistic (*H*)	*I*^2^ statistic (*I*^2^)	Cochran’s *Q* test (*Q*)	*p* value
0	1	0	0.29	0.59

#### Tau-Squared (Tau^2^)

Tau^2^ value reflects the between-study variance in our meta-analysis. Tau^2^ is a useful measure because it is sensitive to the effect size and not sensitive to the number of studies in the analysis. We can infer the present calculated Tau^2^ suggested that the total heterogeneity between studies = 0.

#### *H* Statistic

Within the context of meta-analysis, *H* statistic (*H*) is the proportion of total variability due to heterogeneity. *H* does not depend on sample size and theoretically it ranges from a lowest value of one to infinity. A value of *H* = 1 represents complete homogeneity. In the present study, *H* = 1.

#### *I*^2^ Statistic (*I*^2^)

The *I*^2^ statistic is a descriptive value and not a measure of the real variation across the underlying true effects. Its scale ranges from 0 to 100%. *I*^2^ statistic is a choice test to quantify heterogeneity and calculates the percentage of variation across studies that is due to heterogeneity rather than chance. The present result of *I*^2^ = 0% indicated that all variability in effect size estimates is due to sampling error (chance) and does not require further explanation.

#### Cochran’s *Q* Test (*Q*)

The purpose of the Cochran’s *Q* test (*Q*) statistic is to demonstrate heterogeneity but does not reveal its extent. It only indicates the statistical significance. The *I*^2^ statistic quantifies the magnitude of such heterogeneity. *Q* value is used to determine if variations are due to chance or they are due to differences between studies. *Q* is also called chi-square test for heterogeneity or homogeneity. A weakness of the *Q* statistic is its reduced power to detect true heterogeneity among studies when the meta-analysis includes a few studies. The present replication study is based on two studies. As a counterweight for the low power of *Q*, we opted to test for heterogeneity using an alpha level of 0.10, rather than 0.05, which increases the probability of finding heterogeneity. In the present study, the observed value for *Q* = 0.29; *p* value 0.59. Since the observed *p* value was higher than 0.10, then we assume variations are due to chance and not differences between studies.

## Discussion

The strong association between AQP1 gene sequence variant rs1049305 (C to G) and marathon running is impressive, and we can infer that it promotes whole-body fluid regulation that affects many aspects of performance level. Regulation of water flow across cell membranes is essential for maintaining an appropriate fluid balance within the cell. Physical activity and exercise disturb muscle cell homeostasis. Depending on the exact nature of the stress, skeletal muscle may adapt by increasing size, improving neuromuscular performance, or increasing endurance [21]. If each cell is to complete the reactions demanded for marathon running performance at optimal speed, movement, and endurance, the body must have adequate access to fluid [22]. AQP1 is present in the plasma membrane of muscle fibers and promotes water transport between the blood and myofibrils during muscle activity [23]. This water transport is essential since some of the functions of water include transporting nutrients and aiding in their digestion and absorption, repairing and replacing old tissues, flushing the system of toxic wastes, maintaining a constant body temperature through sweating, thermal capacitance, and blood flow regulation as well as lubricating the joints and tissues [24].

An adequate body fluid balance may improve blood flow through the smallest vessels, which will enhance oxygen delivery to the working muscles [25]. Metabolic adaptations in skeletal muscle, such as optimizing fluid balance, are critical for marathon running performance. Deficiencies in cell fluid management are likely detrimental to marathon running capacity and performance level. Therefore, the fine regulation of water flow by the AQP1 channel as influenced by DNA sequence variations in the AQP1 gene could explain the observed differences in the genotype prevalence between fast and slow marathon runners.

Replication of findings from genetic association studies is a fundamental assessment process in research endeavors leading to new knowledge in genetic epidemiology and related scientific fields. The main functions of replication are the verification of a research finding or piece of knowledge, control of sampling error, control of artifacts, control of fraud, the generalization of results to a different or broader population, and the verification of the initial research hypothesis [26, 27]. The aim is to obtain the consistent results. The process of acceptance of new knowledge considers the reproducibility of new findings rather than the “authority of their originators” [26]. Some have suggested that the criteria for a successful replication study should include the same genetic variant, the same direction of the association, the same definition of the phenotype, and the same ethnic group as reported in the original study [14, 15]. Other factors include the assurance of adequate statistical power, a sound epidemiological design aimed at minimizing biases, highly trained technicians, and topic area familiarized investigators.

Some have argued that there is no such thing as an exact replication [26]. The main argument is there are always differences between the original study and the attempted replication. For instance, the date, setting of the experiment, the experimenters, ethnic group, minor differences in reagents, and the execution of experimental protocols, these differences are classified as obvious or subtle. Therefore, efforts to repeat a methodology do not represent an exact replication [26].

The main finding of the present study was the replication of the association between the DNA sequence variant rs1049305 (C > G) in the 3′UTR of the AQP1 gene and MR performance level in Hispanic male marathon runners. The present exact replication process observed the same rigor, methods, means, ways, ethnic group, and personnel as described in the original study [5]. Compliance with the latter observations is critical in the exact replication of genetic associations [13, 15]. Previous studies [8, 9] provided support to the notion that the AQP1 gene could be part of a highly intricate network of molecular, cellular, and biological components that trigger functions and processes specific to CE running performance in humans. Perhaps the extreme complexity of such network interaction, within the human biology and environment variability domain, had been a factor in the lack of consistent results examining candidate genes by others [28, 29], while examining the association of genetic markers and MR as well as endurance performance domain across populations.

Replication or corroboration studies using comparable subjects and research design as those used in the initial study are rarely conducted in exercise and sports science research. Replication and corroboration attempts have violated the principle of specific physiological adaptations by training modality and or sports discipline. For instance, attempts to replicate genetic associations with candidate genes observed in endurance modalities (e.g., long-distance running) by examining subjects in team sports or activities with different metabolic demands. In the latter cases, such factor could lead to failure to replicate or even corroborate findings [28, 29]. Failure to replicate or corroborate findings is not necessarily an indicator for rejecting the hypothesis of genetic associations. The literature is rich in those arguments [30–33].

The present replication study assessed the validity of the findings of Martinez el al [5].. To enhance the power of comparisons, we performed a meta-analysis combining the results of both studies in addition to performing individual study comparisons. This approach provided four indicators of heterogeneity, which is a critical factor leading to the validation of our results. Factors like statistical power and sample size are always an issue in genetic epidemiology studies and in other fields. Within the limitations of the present replication study, we can argue that the findings are robust.

## Conclusion

The exact replication of the initial findings of Martinez et al. [5] is highly relevant and provides strong support for an association between the AQP1 gene and MR performance level. The findings further contribute to the body of information suggesting the possibility that AQP1 or close molecular genetic mechanism is part of the complex biological processes that determines MR performance level and long-distance running performance. The greater sample size in the present replication study versus the original publication (*∆* = 646) results in a greater statistical power than in the initial study. This study examined a highly specific human sample and a favorable statistical power. We are in the early stages of deciphering the complex polygenic biology (molecular, biochemical, physiological, and mental) underlying MR performance level. The study of AQP1 deserves further detailed systematic scrutiny.

## Data Availability

The datasets used and/or analyzed during the current study are available from the corresponding author on reasonable request.

## Abbreviations

3′UTR: 3′ Untranslated region
AQP1: Aquaporin-1 channel
AQP1: Aquaporin-1 gene
AQPs: Aquaporins
C > G: C to G nucleotide change
CC: Homozygous for C-allele
CG: Heterozygous for C-allele
CI: Confidence interval
CO_2_: Carbon dioxide
DNA: Deoxyribonucleic acid
FE: Fixed effect model
GG: Homozygous for G-allele
*H*: Heterogeneity
HgCl_2_−: Magnesium chloride
*H*_i_: Heterozygosity index
HWE: Hardy-Weinberg equilibrium
*I*^2^: *I*^2^ statistic
MR: Marathon running
OR: Odds ratio
PCR: Polymerase chain reaction
PIC: Polymorphism information content
*Q*: Cochran’s *Q* test
rs: Reference single nucleotide polymorphism cluster identification number
SNPs: Single nucleotide polymorphisms
Tau^2^: Tau-squared statistic
